# A case of spontaneous pneumothorax due to paragonimiasis in North America with literature review

**DOI:** 10.1016/j.idcr.2023.e01742

**Published:** 2023-03-09

**Authors:** Seung Ah Kang, Parag Kumar Patel, Sachin Patil, Andres Bran-Acevedo, Lester Layfield, Sebastian Wiesemann, William Roland

**Affiliations:** a M4 Medical student, University of Missouri School of Medicine, Columbia, USA; b Department of Medicine, Division of Infectious Diseases, University of Missouri, Columbia, USA; c Department of Medicine, Division of Pulmonary and Critical Care Medicine, University of Missouri, Columbia, USA; d Department of Pathology and Anatomical Sciences, University of Missouri, Columbia, USA; e Department of Cardiothoracic Surgery, University of Missouri, Columbia, USA; f University of Missouri Hospital and clinic, 1 Hospital Dr, Columbia, MO 65212, USA

**Keywords:** *Paragonimus kellicotti*, Paragonimiasis, Parasitic lung infection, Lung fluke, North America

## Abstract

The species, *Paragonimus kellicotti* , causes human paragonimiasis in North America. As a foodborne disease, human infection with *P. kellicotti* occurs after eating raw or undercooked crayfish containing metacercariae. Many risk factors have been described in the literature, including young adult age, male, alcohol consumption, outdoor activities involving rivers within Missouri, and ingesting raw or partially cooked crayfish. Here, we report a case of a 41-year-old male with a 5-year history of cough who presented with acute shortness of breath. Further workup showed mild eosinophilia and spontaneous pneumothorax. A definitive diagnosis was made with a lung biopsy, which showed *P. kellicotti* eggs. Further questioning revealed that the patient took a hunting and river rafting trip on a river in Missouri 5 years ago, though the history was negative for any crayfish consumption. Paragonimiasis should be considered in those with associated clinical features, including cough and eosinophilia, with a history of a river raft float trip in Missouri, even if the history is negative for crayfish ingestion or travel.

## Introduction

Paragonimiasis is a parasitic infection caused by the lung fluke, *Paragonimus*. It has a global distribution, most notably in Southeast Asia and China [1], [2]. *Paragonimus kellicotti* species are endemic to North America [3]. Cases of paragonimiasis within the United States are sporadic, but autochthonous infections due to *P. kellicotti* have been increasing in the Midwest [4]. We describe a case of a young male with spontaneous left-sided pneumothorax due to *P. kellicotti* infection without a history of crayfish ingestion or Southeast Asia travel.

## Case report

**Fig. 1:**
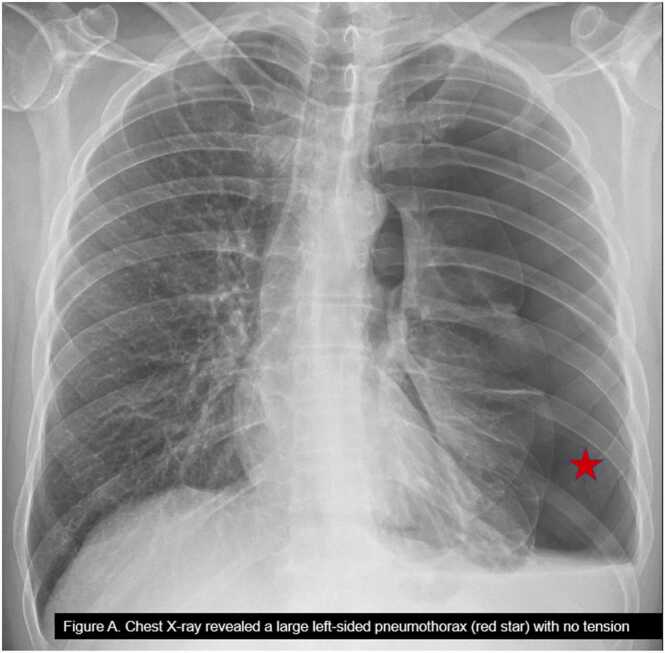
Chest X-ray revealed a large left-sided pneumothorax (red star) with no tension.

A 41-year-old male with no prior medical history presented to an urgent care clinic at an outside hospital (OSH) with dyspnea, worse on exertion, occasional wheezing for three weeks, and intermittent diarrhea for the last six months. He also admitted to a chronic nonproductive cough for the previous five years and a recent streptococcal throat infection in his daughter. He was an active smoker with 30 packyears of smoking and occasional marijuana and beer intake. His family history was significant for lung cancer in his father. Clinical examination revealed mild tachycardia of 109 beats per minute and an absence of lung sounds on the left side. Chest X-ray (CXR) two views revealed a large left-sided pneumothorax with no tension (Fig. 1). He was immediately referred to the emergency department at OSH, where he underwent placement of a 20 French chest tube insertion into the left fourth intercostal space and was admitted to the surgical service. A repeat CXR confirmed the position. Complete blood cell count with differential (CBCD) revealed elevated eosinophils at 5.9 % and an absolute eosinophil count (AEC) of 610.7 cells/µL, whereas the complete metabolic panel was normal. Three hours later, a repeat CXR revealed mild improvement of the pneumothorax with a concern for slow re-expansion.

**Fig. 2:**
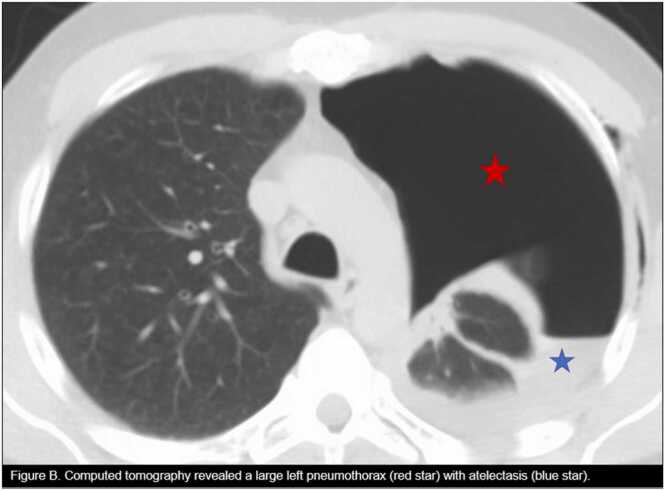
Computed tomography revealed a large left pneumothorax (red star) with atelectasis (blue star).

Computed tomography (CT) scan of the chest displayed the left-sided chest tube outside the pleural space (Fig. 2). The interventional radiology (IR) team then placed a 14-French chest tube in the pleural space with pneumothorax improvement. CXR on the following day revealed the left chest tube in position and a tiny apical pneumothorax. Due to the significant improvement in pneumothorax, the primary team removed the left chest tube with a follow-up CXR, revealing a small apical left pneumothorax. A CBCD on day three showed elevated eosinophils of 11.3 % with an AEC of 1210.2 cells/µL. CXR on day three revealed an increasing pneumothorax, and the IR team placed a 10-French left pleural chest tube connected to a heimlich valve. A follow-up CXR confirmed the position and pneumothorax resolution. He was discharged to home on day four with empirical oral Keflex 500 mg twice a day for five days. After three days, he returned to the outpatient clinic, and a CXR post-clamping of the chest tube demonstrated an increase in left pneumothorax. The chest tube was then unclamped, recanted to the heimlich valve, and he was referred to our academic center's cardiothoracic surgery (CTS) clinic.

After two weeks, he was seen at the CTS and admitted to improvement in his symptoms with the chest tube draining clear yellow drainage of 30 mL/day. Clinical examination revealed stable vital signs. The heimlich valve was removed, and the chest tube was connected to a Pleur-evac to evaluate for persistent pneumothorax. Bubbles were observed in the atrium, demonstrating persistent air leak. The primary impression was of a primary spontaneous pneumothorax with persistent air leak for the last three weeks. He was scheduled for a Video-assisted thoracoscopic surgery (VATS) pleurodesis via pleurectomy and likely wedge resection of the lung bulla the next day. He underwent preoperative flexible bronchoscopy, which was normal. He underwent left VATS with an intercostal nerve block (Bupivacaine plus Exparel), pleural lysis, intrapleural fibrin removal, therapeutic wedge resection(Left lower lobe [LLL], Left upper lobe [LUL]), partial pleurectomy and placement of a 24-French chest tube under general anesthesia. Left anterior pleural fluid and fibrin samples were sent for culture. LLL and LUL wedge specimens were sent to pathology along with the left parietal pleura. Post-operatively he was awakened from general anesthesia, extubated, and taken to the recovery unit in stable condition.

**Fig. 3:**
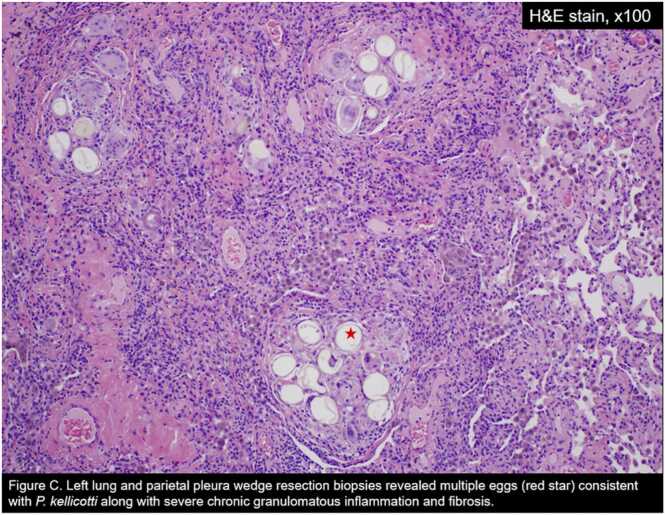
Left lung and parietal pleura wedge resection biopsies revealed multiple eggs (red star) consistent with *P. kellicotti* along with severe chronic granulomatous inflammation and fibrosis.

**Fig. 4:**
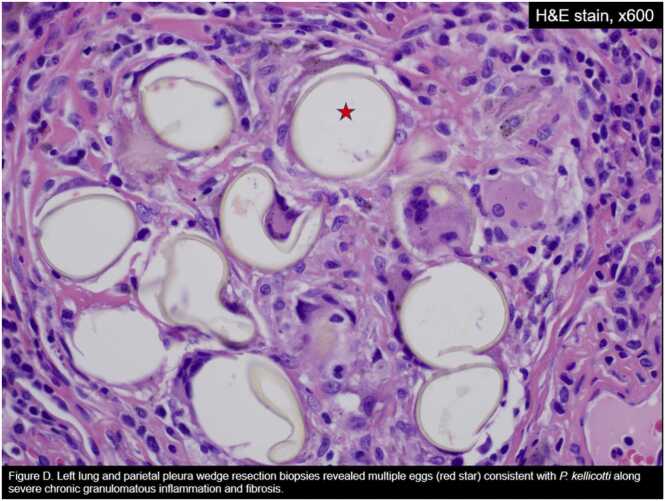
Left lung and parietal pleura wedge resection biopsies revealed multiple eggs (red star) consistent with *P. kellicotti* along severe chronic granulomatous inflammation and fibrosis.

**Fig. 5:**
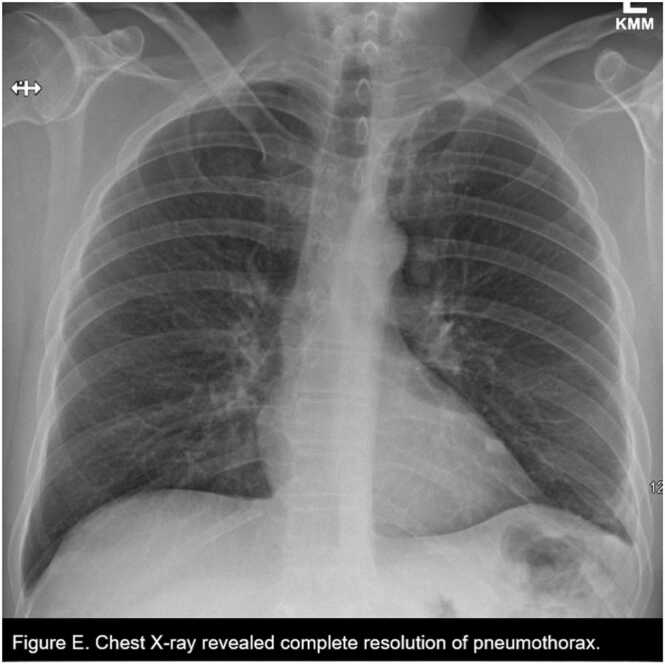
Chest X-ray revealed complete resolution of pneumothorax.

The following day, a CBCD revealed a normal AEC (14.2 cells/µL), and he was discharged home with a left surgical chest tube attached to a Pleur-evac water seal with plans to follow up at the clinic in one week for reassessment. Intraoperatively significant inflammation was observed at the site of biopsies, and he was discharged on a 7-day course of oral Augmentin. LLL and LUL wedge resection and left parietal pleura histopathology revealed multiple parasite eggs (consistent with *Paragonimus kellicotti*), severe chronic granulomatous inflammation with fibrosis, negative for malignancy and changes consistent with pneumothorax (Fig. 3, Fig. 4). The CTS team was informed of the pathological findings immediately, and an Infectious Disease (ID) team referral was placed. The patient was started on praziquantel 600 mg orally three times a day for three days and recommended to follow up at the ID clinic in three weeks. At the one-week CTS clinic follow-up, he had completed the praziquantel course with minimal chest tube output (100 mL /day) over the last few days. The Pleur-evac was connected to suction to remove any additional air. No expiratory air leak was noted with coughing or forced expiration. The chest tube was removed and sutured down. At three weeks ID clinic and CTS clinic follow-up, he had no new symptoms, a repeat CXR two views (Fig. 5) revealed no pneumothorax, the human immunodeficiency virus serological study was negative, the chest tube surgical site was clean, and the sutures were removed.

Further history revealed that the patient had lived in Missouri for most of his life. He worked at his own moving company. He recalled going on a hunting and river rafting trip five years ago along the Missouri river, during which he consumed alcohol and wild boar meat cooked overnight. On this trip, his wife and friends ate cooked crayfish, and he denied any crayfish intake. Three months after this trip, he recalled having a productive cough with minimal clear white phlegm and hemoptysis for about 12 months. All symptoms resolved except for cough, which he attributed to his chronic tobacco use.

## Discussion

Human paragonimiasis is endemic to tropical and subtropical regions in East and Southeast Asia, West Africa, and the Americas, with the most significant risk of infection occurring throughout Asia [1], [2]. *P*. *westermani*, *P*. *heterotremus*, and *P*. *skrjabini*/*P*. *miyazakii*, distributed in Asia, are the most important species as the cause of paragonimiasis [1]. *P*. *africanus* and *P*. *uterobilateralis* occur in Africa, mainly in the rainforests of West and Central Africa [3]. *P*. *mexicanus* occurs in Central and South America [4]. In North America, human paragonimiasis is caused by the species *P*. *kellicotti* [5]. Human infection occurs after consuming raw or partially-cooked crabs and crayfish and rarely after eating raw meat of a paratenic mammal host [2]. As a foodborne disease, human paragonimiasis risk is related to local dietary preferences, practices, and customs and is influenced by the globalization of food products [1].

An understanding of the intricate life cycle of *Paragonimus* helps explain disease transmission and clinical manifestations. First, eggs hatch in fresh water and release motile miracidia that infect the first intermediate hosts (snail species) [6]. Miracidia develop into motile cercaria that invade the tissues of the second intermediate host (crustaceans, such as crabs or crayfish). Here, cercaria develop into metacercariae. Infection of the definitive mammalian hosts (humans, canines, and felines) occurs after ingesting a crustacean containing metacercariae. Metacercariae then invade the host's small intestine, enter the peritoneal cavity, migrate through the diaphragm, and into the pleural space, waiting for another metacercaria to pair with. Next, the immature adult mates and penetrate the lung parenchyma, form a fibrous cyst, and lay eggs. Eggs are released into the respiratory airways and voided in either sputum or passed in the feces if swallowed. The release of eggs into the environment completes the life cycle of *Paragonimus*. Metacercariae in a paratenic host, usually a mammal, remain latent in the host's muscles as they cannot develop into sexually competent adults. The definitive host can also acquire paragonimiasis after consuming a paratenic host containing latent juvenile metacercariae as they continue their lifecycle and become mature adults [6]. Paratenic hosts for paragonimiasis in Japan have been described and include wild boar and sika deer [2].

Since raw or undercooked crayfish are not a part of the North American diet, human infection with *P. kellicotti* is rare. From 1969–2017, 21 cases of human paragonimiasis due to *P. kellicotti* were reported in the literature [7]. Of these, the majority were associated with young adult age (18–39 years), male sex, and 15 alone were reported in Missouri [7]. Nearly all individuals with *P. kellicotti* infection have been reported to eat raw or incompletely-cooked crayfish [7]. Most crayfish consumption occurred after alcohol intoxication while on float (recreational river) trips, canoeing, and camping [8], [9], [10], [11]. Since alcohol intoxication may impair judgment and relax normal dietary inhibitions, alcohol consumption at the time of crayfish ingestion is a significant risk factor [4], [5], [12]. Other situations that led to crayfish consumption include being prompted by dares and showing outdoor survival skills to others.

In our case, the patient had multiple risk factors for *P. kellicotti* paragonimiasis, including his relatively young age of 41 years, male sex, presence of an outdoor recreational trip in a river in Missouri, and alcohol consumption. Although he noted that his wife and friends ate cooked crayfish, the patient denied any crayfish intake on this trip. He recalled consuming wild boar meat cooked overnight. Human cases of *P. kellicotti* infection acquired through a paratenic host have not been reported in the literature, though they are likely to be present in nature [6]. One case report described *P. westermani* infection in a woman who denied freshwater crayfish or crab consumption but had a history of preparing raw freshwater crabs for several decades [13]. The authors suspected that her hands, chopping boards, or utensils were contaminated with metacercariae. The transmission mode remains unclear in our patient, with the possibilities being crayfish stored in the same cooler as beer, contaminated utensils, or hands of others consuming crayfish or inadequately cooked wild boar meat containing metacercariae.

The pleural and pulmonary features of *P. kellicotti* paragonimiasis are similar to *P. westermani* paragonimiasis found in Asia [5], [10]. Early-stage disease, or acute paragonimiasis, occurs between the initial infection and fluke migration into the pleural space [6]. Days to weeks after consuming raw or undercooked crustaceans, patients are usually asymptomatic, but those with heavy worm burdens may experience fever, abdominal pain, and diarrhea [1], [10]. As larvae migrate within the pleural cavity, pleuritic chest pain and pleural effusion may develop [6]. In *P. kellicotti* infection, the incubation period ranges from 14 to 75 days, and common symptoms at presentation include cough, fever, fatigue/malaise, and weight loss [7]. The vast majority of patients also have peripheral eosinophilia [7].

Late-stage disease, also known as chronic pleuropulmonary paragonimiasis, refers to the period when parasites from the pleura migrate to the location of cyst formation [6]. The ideal place for cyst formation is in the lung parenchyma near the airway so that the eggs can exit through respiratory secretions. In parenchyma-based disease, the most common symptoms are cough, hemoptysis, and dyspnea [1]. Rarely, cysts may be produced near the pleura resulting in a release of eggs into the pleural space leading to an end to its life cycle [6]. The inflammation and fibrosis that follows in pleura-based disease can lead to pleural effusions and pneumo- or hemothorax. Common radiologic features include lung consolidation, pleural effusions, cystic lesions, linear streaks, nodules, pleural thickening, ring shadow, and calcified lesions [6]. Linear, band-like opacities running differently from the bronchovascular bundles represent migration tracts of worms in the lung parenchyma that strongly support the paragonimiasis diagnosis [14]. An extrapulmonary disease with *Paragonimus* species may also occur if immature flukes migrate to ectopic locations, including the brain and skin [10]. *P. kellicotti* has been shown to cause cerebral paragonimiasis [12], [15].

A definitive diagnosis is made with the microscopic detection of eggs in sputum or feces; eggs may also be found in bronchoalveolar (BAL) fluid, pleural effusion fluid, and surgical specimens like lung biopsies [1], [2]. Serological testing, such as Enzyme-linked immunosorbent assay (ELISA), is recognized as a more reliable method for diagnosis due to its high sensitivity and high specificity [2], [16]. Because trematode infections in humans are rare in North America, cross-reactivity of antibodies across different trematode species (schistosomiasis, fascioliasis, clonorchiasis) is less of a concern for the serodiagnosis of *P*. *kellicotti* [5]. Paragonimiasis is commonly misdiagnosed as culture-negative pulmonary tuberculosis due to the similarity of symptoms, radiographic features, and geographic distribution [17]. Therefore, diagnosis may be delayed by months or years, with some patients being subjected to expensive and ineffective treatments [5].

Praziquantel is the treatment of choice for paragonimiasis, regardless of the species [2]. The recommended dose is oral 25 mg/kg three times a day for three days, with excellent cure rates [18]. Triclabendazole is similarly effective (10 mg/kg oral once or twice) and may be better tolerated than praziquantel, though its efficacy on cerebrospinal paragonimiasis is undetermined [18], [19]. Abnormal migration leading to extrapulmonary disease is more likely to occur in heavy infections, highlighting the importance of early detection and appropriate treatment [6].

## Conclusion

In this case report, we highlight *P. kellicotti* as an emerging pathogen and aim to increase awareness among providers. Information on the risk factors for *Paragonimus* infection, such as residence, travel history, and dietary habits, is helpful for the diagnosis. A presentation of cough, fever, hemoptysis, and eosinophilia, in addition to a reported history of a river raft float trip in Missouri, should raise clinical suspicion of paragonimiasis even in the absence of crayfish ingestion or travel.

## Ethics Approval and Consent to Participate

Care was taken to ensure that all patient identifiers were removed in the process of creating this case report, the patient was made aware of this case report.

## Funding

There are no sources of funding to report for this case report.

## CRediT authorship contribution statement

Seung Ah Kang - Principal author Paragkumar Patel - Co-author Sachin Patil - Faculty advisor and contributor Bran Andres Acevedo - Faculty advisor and contributor Lester Layfield – Faculty advisor and contributor Sebastian Wiseman - Faculty advisor and contributor William Roland - Faculty advisor and contributor.

## Conflicts of Interest

The authors declare that they have no conflicts of interest.
